# Genetic diversity and distribution of *Senegalia senegal* (L.) Britton under climate change scenarios in West Africa

**DOI:** 10.1371/journal.pone.0194726

**Published:** 2018-04-16

**Authors:** Paul Terwase Lyam, Joaquín Duque-Lazo, Walter Durka, Frank Hauenschild, Jan Schnitzler, Ingo Michalak, Oluwatoyin Temitayo Ogundipe, Alexandra Nora Muellner-Riehl

**Affiliations:** 1 Department of Molecular Evolution and Plant Systematics & Herbarium (LZ), Institute of Biology, Leipzig University, Germany; 2 National Centre for Genetic Resources and Biotechnology, Ibadan, Nigeria; 3 German Centre for Integrative Biodiversity Research (iDiv) Halle-Jena-Leipzig, Germany; 4 Department of Forest Engineering, Laboratory of Dendrochronology, Silviculture and Global Change, DendrodatLab-ERSAF, University of Cordoba, Campus de Rabanales, Córdoba, Spain; 5 Department of Systematic Botany and Functional Biodiversity, Institute of Biology, Leipzig University, Germany; 6 Department of Community Ecology (BZF), Helmholtz Centre for Environmental Research–UFZ, Halle, Germany; 7 Department of Botany, University of Lagos, Akoka, Lagos, Nigeria; National Cheng Kung University, TAIWAN

## Abstract

Climate change is predicted to impact species’ genetic diversity and distribution. We used *Senegalia senegal* (L.) Britton, an economically important species distributed in the Sudano-Sahelian savannah belt of West Africa, to investigate the impact of climate change on intraspecific genetic diversity and distribution. We used ten nuclear and two plastid microsatellite markers to assess genetic variation, population structure and differentiation across thirteen sites in West Africa. We projected suitable range, and potential impact of climate change on genetic diversity using a maximum entropy approach, under four different climate change scenarios. We found higher genetic and haplotype diversity at both nuclear and plastid markers than previously reported. Genetic differentiation was strong for chloroplast and moderate for the nuclear genome. Both genomes indicated three spatially structured genetic groups. The distribution of *Senegalia senegal* is strongly correlated with extractable nitrogen, coarse fragments, soil organic carbon stock, precipitation of warmest and coldest quarter and mean temperature of driest quarter. We predicted 40.96 to 6.34 per cent of the current distribution to favourably support the species’ ecological requirements under future climate scenarios. Our results suggest that climate change is going to affect the population genetic structure of *Senegalia senegal*, and that patterns of genetic diversity are going to influence the species’ adaptive response to climate change. Our study contributes to the growing evidence predicting the loss of economically relevant plants in West Africa in the next decades due to climate change.

## Introduction

Biodiversity is crucial to human well-being as it provides fundamental ecosystem services. At the same time, biodiversity is under threat by human population growth, increased land use, and CO_2_ emissions, which are all direct or indirect drivers of climate change [1–3]. One of the regions most threatened by climate change is sub-Saharan Africa [4, 5]. This part of Africa is a developing region where deforestation and desertification are of major concern. Within sub-Saharan Africa lies the so-called arid and semi-arid land traversing the savannah belts of the region and making up the Sudano-Sahelian zone (SSZ) [6]. This region is socio-economically and ecologically important, but at the same time threatened by climate change, land degradation due to unsustainable agriculture, deforestation, and overgrazing [7].

The effect of environmental and climate change has been studied for a few African plant species [3, 8, 9]. Mostly, such studies focussed on predicting the impact on ecosystems and species [10]. These studies are highly valuable, but do not account for intra-specific genetic diversity [11]. Global studies indicate that large proportions of suitable habitat and the respective species will be lost during this century due to climate change [3, 11, 12]. However, climate change will also affect intraspecific genetic diversity [13]. Studying the effects of climatic alterations on genetic diversity is necessary if we are to understand the evolutionary consequences of climate change and its long-term effects on species distribution [14]. To assess future impacts on biodiversity many studies have used the Intergovernmental Panel on Climate Change’s (IPCC) scenarios from the Special Report on Emission Scenarios, which project emissions and socio-economic changes [10, 15]. The latest set of climate model simulations assumes four different sets of possible futures. They are known as the Representation Concentration Pathways (RCPs) representing low (RCP 2.6), medium (RCPs 4.5 and 6.0) or high (RCP 8.5) emissions [16, 17].

In the sub-Saharan zone, one important species is *Senegalia senegal* (L.) *Britton*. (Fabaceae), syn. *Acacia senegal* (L.) Willd. [18]. The species, which is known for its exudate named ‘gum arabic’, is a small deciduous tree, native to arid and semi-desert regions of sub-Saharan Africa, but has also been introduced in other regions (e.g. the Indian sub-continent) [19, 20]. In western Africa, *S*. *senegal* naturally occurs either as a dominant extensive pure stand or in co-habitation with other species in a variety of vegetation types, including semi-desert grassland, *Anogeissus* woodland and rocky hill slopes [21]. The species can grow on sandy, skeletal and slightly loamy soils. However, it shows preference for coarse-texture soils such as fossil dunes, with a soil pH of 5 to 8. Although *S*. *senegal* has been observed to grow in areas that receive 100 to 950 mm annual rainfall, it thrives best in areas with 300 to 400 mm annual rainfall. The species can tolerate five to 11 months of drought and can survive temperatures high as 43°C, dry wind and sandstorms, but is highly sensitive to frost. The altitude ranges from 100 to 1700 metres above sea level within the African ASAL. The species is insect-pollinated, and predominantly out-crossing [7, 22]. Seeds are dispersed at least partly by animals, especially ungulates [23]. The astringent, emulsifying, film-forming and encapsulating properties of the gum arabic represents an important economic resource, which is often used in the food-processing, pharmaceutical, cosmetic, and lithographic ink industries [24–27]. In addition to playing a preferential role in the context of land degradation and desertification control, *S*. *senegal* has been reported to be resilient to drought and grazing making it an important species in agroforestry systems [28]. The current conservation status of the species is “least concern” (IUCN). However, field observations show that the species is locally under intense pressure from animal grazing, pest and human activities (personal observation) thus corroborating the earlier report of Eisa et al. [29]. In addition, climate change will likely further increase the vulnerability of the species through disturbances of natural habitat. As a consequence, *in situ* conservation and future utility of *S*. *senegal* could be jeopardized [30, 31], with obvious repercussions for human livelihoods. Various aspects including soil physicochemical properties [30], pollination and breeding systems [32], ecology [27, 33], phylogeography [23], and population genetics [34, 35] in *S*. *senegal* have been previously investigated. In *S*. *senegal*, intraspecific genetic variation has been studied both on regional levels [34, 35] and for range-wide phylogeography [23]. The latter shows a recent range expansion to west Africa with genetic diversity reported to be low in the region [23]. However, no detailed analyses are available for the SSZ of West Africa where the available phylogeographic analysis is characterized by sampling gaps.

This study aims to fill existing sampling gaps and identify genetic diversity and population structure of *Senegalia senegal* (L.) *Britton*. in the SSZ of West Africa. We used both nuclear and maternally inherited plastid microsatellite markers. Furthermore, we used species distribution modelling to forecast the potential range loss and loss of intra-specific genetic diversity under climate change and promote strategies towards effective management and conservation of *S*. *senegal* in the West African SSZ. Specifically, we address the following questions: 1) Is the genetic diversity in the West African SSZ as low as previously stated for the region? 2) How is genetic diversity structured within the West African SSZ? 3) How would future climate impact the distribution of *S*. *senegal* in the West African SSZ? We discuss the potential impact of climate driven range changes of *S*. *senegal* in the SSZ of West Africa on extant genetic variation.

## Materials and methods

### Ethic statement

No specific permissions were required for sampling of leaf material. There is no previous report stating that *Senegalia senegal* is threatened or under protection in any of the sampled locations. According to IUCN, the species has “least concern” conservation status.

### Study area and sampling

**Fig 1:**
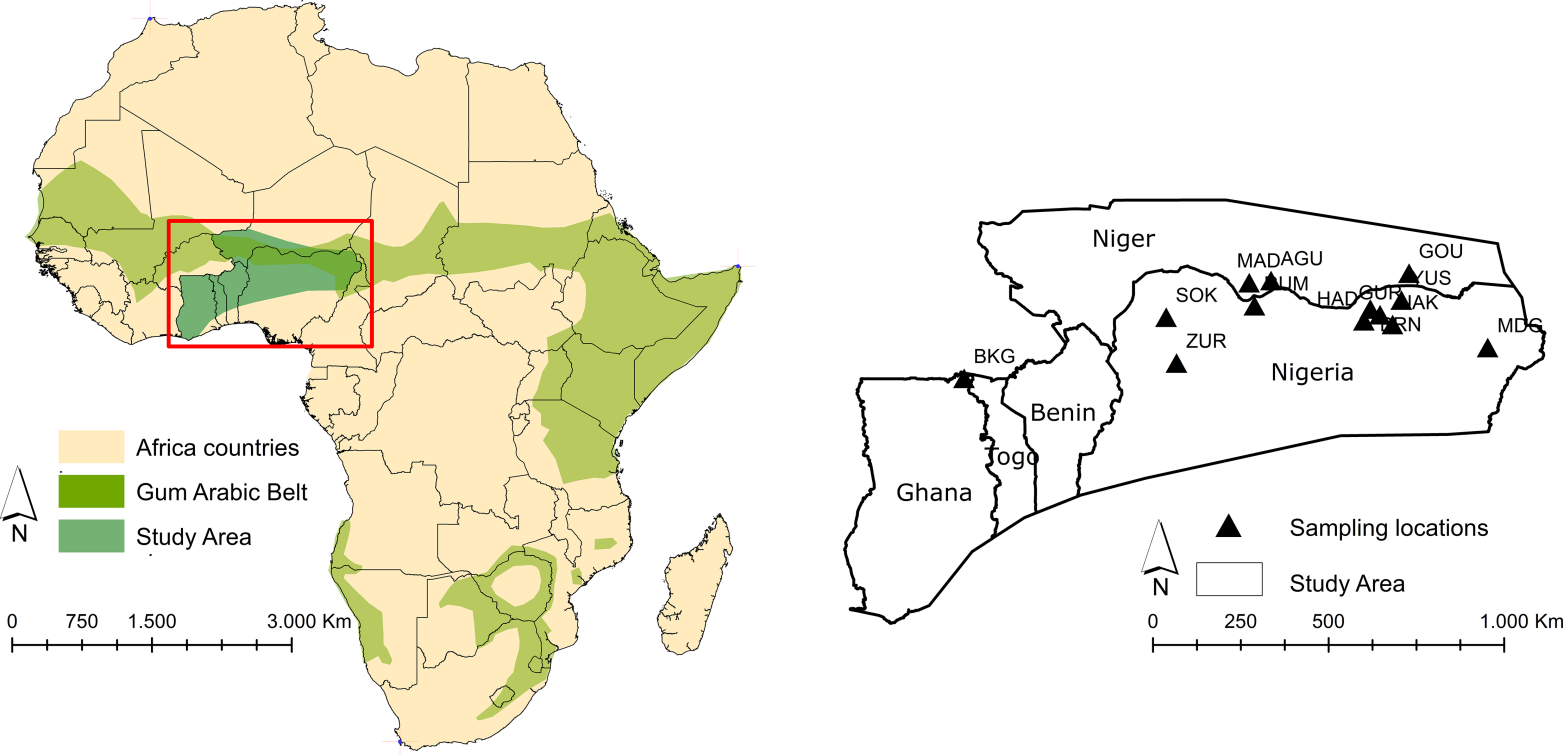
Location of thirteen *Senegalia senegal* populations sampled from West Africa.

Fresh leaf samples of *Senegalia senegal* were collected from thirteen localities along the fringes of the gum arabic belt, situated between latitude 11°N and 13°N and longitude -0.1°W and 13.2°E between 2012 and 2016. The study area is a mixture of tropical woodland, grassland savannah and semi-desert steppe that transverses the SSZ and extends eastwards from north-eastern Ghana to north-eastern Nigeria (Fig 1). The spatial distances between neighbouring collection sites ranged between 35 km (BIR–GUR) and 1,500 km (BKG–MDG). The sample size per site was between 13 and 32 individuals, with a total of 316 samples. Within sites distance was between 10 m and 10 km, depending on the size of the population. Details of parameters sampled from the sites are shown in Table 1. Field-collected material was dried in silica gel prior to DNA extraction [36]. At least one individual per population was deposited as a voucher specimen at both, the herbaria of the National Centre for Genetic Resources and Biotechnology (NACGRAB), Ibadan, Nigeria, and Leipzig University (LZ), Germany.

**Table 1:** Sampling data for *Senegalia senegal* plant material analysed in this study.

N0.	Site code	Latitude	Longitude	N	Altitude (m)	Annual Rainfall (mm)	Mean annual Temp (°C).	Location—Country
1	BKG	11.015	-0.199	13	254	800–1000	28	Bawku, Ghana
2	ZUR	11.407	5.239	20	395	800–1000	27	Zuru, Nigeria
3	SOK	12.578	4.974	22	272	700–800	28	Sokoto, Nigeria
4	MAD	13.461	7.102	32	364	400–500	27	Madarounfa, Niger
5	AGU	13.517	7.662	28	438	400–500	27	Aguie, Niger
6	RUM	12.874	7.236	26	484	500–600	27	Rumah, Nigeria
7	HAD	12.487	10.042	22	356	500–600	28	Hadejia, Nigeria
8	BRN	12.787	10.205	22	347	400–500	28	Birninwa, Nigeria
9	GUR	12.642	10.453	21	350	400–500	28	Guri, Nigeria
10	JAK	12.391	10.775	22	350	500–600	28	Jakusko, Nigeria
11	GOU	13.706	11.196	25	344	200–300	28	Goudoumaria, Niger
12	YUS	12.892	11.150	26	341	300–400	27	Yobe, Nigeria
13	MDG	11.802	13.211	24	324	500–600	27	Maiduguri, Nigeria

Abbreviations of populations are listed in the first column; number of samples per population (N). Mean annual rainfall and temperature sourced from Worldclim 1.4 and 2.0 respectively.

### Microsatellite genotyping

Total genomic DNA was extracted from 30 mg of silica-dried leaves using a Nucleo-spin plant II kit (Macherey-Nagel, Düren, Germany). We used ten nuclear microsatellites (hereafter nSSR) specifically designed for *Senegalia senegal* by Assoumane *et al*. [37], and two universal plastid microsatellites (hereafter cpSSR) designed by Weising *et al*. [38]: ccmp5 and ccmp10. DNA amplification was performed using a multiplex PCR kit (Qiagen, Venlo, The Netherlands). The PCR mix contained a final volume of 10 μl with 5 μl of 2X Qiagen Multiplex PCR Master Mix, 1 μl of Primer mix (0.2 μM of each forward and reverse primers), 1 μl of Q-Solution, 1 μl of RNase free water and 2 μl of template DNA (10 ng/μl). The PCR was initially heated to 95°C for 15 min, followed by a 3-step touchdown cycling programme consisting of denaturation at 95 ^o^C for 15 mins, annealing at 67 ^o^C for 1.5 mins, extension at 72 ^o^C for 1 min, followed by eight cycles of 94 ^o^C for 30 s, 65 ^o^C for 1.5 min with 2 ^o^C decrease at each cycle, 72 ^o^C for 1 min; 24 cycles at 94 ^o^C for 30 s, 51 ^o^C for 1.5 min, 72 ^o^C for 1 min, and a final extension at 60 ^o^C for 30 min. Amplification was carried out separately for each primer pair in a singleplex-PCR and combined for fragment analysis. PCR products were amplified using 6-FAM-, VIC-, NED-, and PET-labelled primers (Applied Biosystems, Foster City, California, USA). The touchdown cycling program was used on a Mastercycler 2050 model thermal cycler (Thermo Fisher Scientific, Waltham, Massachusetts, USA). PCR products were sequenced on an ABI 3730 automated sequencer (Applied Biosystems) using LIZ 500 or LIZ 600. Allelic binning and scoring of genotypes were performed using GeneMapper v.5.0 (Applied Biosystems). Raw data matrices containing allelic information were double-checked for scoring errors. Samples with more than two missing loci were excluded from further analysis. The final data set consisted of 287 samples for nSSR and 303 samples for cpSSR analyses.

### Genetic data analysis

We used the program MicroChecker v.2.2.3 [39] to estimate the frequency of Null alleles across all samples. For the nSSR data set, intrapopulation genetic diversity was estimated as the total number of alleles per locus and per population (NA), the average number of alleles per locus for each population over all loci (A_avr_), the unbiased estimate of expected (H_E_) and observed (H_O_) heterozygosity [40] within populations using Arlequin v.3.5.1.3 [41]. The number of private alleles (A_PRIV_) was estimated using GDA v.1.0 [42]. FSTAT v.1.2 [43] was used to calculate allelic richness (A_R_), thus correcting for different sample sizes, and inbreeding coefficient (F_IS_). The package genepop v.1.2 [44] was used to perform exact tests of Hardy–Weinberg equilibrium (HWE), and F-statistics estimated by Weir and Cockerhan [45]. Population genetic structure was evaluated using a Bayesian clustering approach, implemented in STRUCTURE v.2.3.4 [46]. Individual’s genotype association from 1 to 13 distinct genetic clusters (K), allowing for admixture, was performed 20 times for each K with 500,000 generations as burn-in period, followed by 500,000 iterations of MCMC analysis assuming that the different populations had correlated allele frequencies without using prior information on the sampling locations of each individual [47]. The “Evanno method” [48] was used to estimate the most likely K [48], as implemented in STRUCTURE HARVESTER [49], but we also scrutinized results of other K for biologically meaningful solutions. To estimate population genetic differentiation, an analysis of molecular variance (AMOVA) was performed based on the clusters suggested by the STRUCTURE analysis using GenAlex v.6. [50]. We tested for isolation by distance (IBD) based on the correlation between genetic and geographical distances between populations using a Mantel test as implemented in GenAlex v.6. [50].

For the cpSSR data, HAPLOTYPE v.1.05 [51] was used to estimate the number of alleles at chloroplast SSR loci (N_acpSSR_), number of haplotypes detected in each population (N_b_), effective number of haplotypes (N_e_), genetic diversity (Dv), haplotype richness (H_r_) and the number of private haplotypes (Prv). A parsimony network illustrating genetic relationships between haplotypes was inferred using the software PopArt [52], assuming single-step mutations between alleles. Total genetic variation among samples was calculated using the phi-statistics of AMOVA which was performed using GenAlex [50]. This total variation was partitioned at three levels—within populations (Phi-PT), among populations within clusters (Phi-PR), and among clusters (Phi-RT) [53].

### Distribution modelling

The sampled point locations (n = 316) were initially increased with 30 records from the GBIF database [54]. The dataset was extensively cleaned with doubtful localities and multiple records within a 30 arc-second gridcell (approximately 1x1 km, see below) being removed. The final set of point locations (n = 251) was then used for estimating the potential distribution of the species. Additionally, pseudo-absence data were created with the same number of points as the presence data (n = 251). Pseudo-absences were randomly generated across the study area with a minimum distance of 1.5 km to each other and the presence localities. Equal numbers of presence and pseudo-absence were used as recommended by Liu et al. [55]. Nineteen bioclimatic variables, together with three topographic (DEM; DEM-derived) variables with a spatial resolution of 30 arc-seconds were downloaded from the public WorldClim database [56]. These variables describe interpolated monthly means of climatic/environmental factors. Additional 66 soil variables with a resolution of 250 meters (S5 Table) were downloaded from the online database Soildgrids250m [57]. The complete set of variables was resampled to 30 arc-seconds spatial resolution in the WGS 84 coordinate system.

The impact of climate change on the current distribution of *Senegalia senegal* was assessed using WorldClim’s future climate change scenarios. We selected two future climate change projections (2050 and 2070) from two global climate models (GISS-ER2 [58, 59] and CCSM4 [60], hereafter GCM) and four RCP scenarios [2]. Moreover, topographic and soil variables were considered constant in future scenarios. Distribution modeling (current and future) of *S*. *senegal* was computed with a maximum entropy approach [61] as implemented in MaxEnt v.3.3.3k [62]. Model performance was evaluated by the Area Under the Curve (AUC) of the Receiver Operating Characteristic (ROC) plot. The final model was selected as the one with highest AUC score [63, 64]. Variable selection was performed in two steps: First, collinear variables were deleted stepwise considering a variance inflation factor (VIF) > 10 as critical threshold [65] using the '*usdm'* R package [66], reducing the number of variables to n = 12. Second, the final set of environmental variables was chosen based on the optimization of the AUC value by the Random Forest (RF) model using the '*AUC-RF'* R package [67], resulting in seven environmental predictors for the final model. In order to assess area gains and losses, we re-classified current and future habitat suitability probability scores into binary data by a threshold equal to prevalence [68]. We then estimated the degree of total area change as the percentage of remaining area in comparison with the current distribution. Values above 100% represent expansion (gain of area distribution) and values below 100% represent the diminution of the total extend from the current prediction. In addition, we mapped the changes between the current and future habitat suitability scores of *S*. *senegal* for each 30 arc-second grid cell. Differences were classified as follows: 1) not present, low probability of occurrence in current and future predictions (<75%); 2) area loss, high probability in the current prediction and low in the future; 3) area gain, low probability of occurrence (<75%) in the current situation and high in future predictions (>75%) and 4) remain, high probability of occurrence (>75%) as previously done by [69].

## Results

### Within-Population genetic diversity

In total, 110 alleles were scored across ten nSSR loci for the 287 genotyped individuals (S1 Table). None of the loci exhibited null allele presence. At the population level, observed and expected heterozygosity values for nSSR markers ranged from 0.42 to 0.73 (mean 0.61) and 0.43 to 0.63 (mean 0.56), respectively. Allelic richness ranged from 2.86 to 4.55 with a mean of 3.71. Private alleles were identified in eleven populations, with GOU having the highest number of four private alleles. Significant deviations from HWE were detected at seven sites with heterozygote deficiency in one site (HAD) and heterozygote excess in six sites (Table 2) while measures of inbreeding coefficient (Fis) were generally negative or very low ranging from -0.28 to 0.12 (mean = -0.06).

**Table 2:** Genetic characteristics of 13 populations of *Senegalia senegal* revealed by ten nuclear and two chloroplast markers.

nSSR								cpSSR						
Population	N	A_R_	NA	A_PRIV_	^+^Ho	^+^He	F_is_^1^	N	N_acpSSR_	N_b_	(N_e_)	Prv	Hrs	Dv
BKG	13	3.598	3.7	2	0.68 (0.27)	0.57 (0.18)	-0.11*	13	2	1	1	0	0	0
ZUR	17	2.856	2.9	1	0.73 (0.21)	0.51 (0.21)	-0.283***	20	2	1	1	0	0	0
SOK	22	3.951	4.2	2	0.67 (0.23)	0.60 (0.15)	-0.119**	22	2	1	1	0	0	0
MAD	30	4.546	5.8	3	0.61 (0.25)	0.60 (0.22)	-0.002	32	2	1	1	0	0	0
AGU	26	3.66	3.9	0	0.64 (0.23)	0.63 (0.22)	0.009	28	2	1	1	0	0	0
RUM	26	3.751	4.6	3	0.54 (0.19)	0.54 (0.15)	0.03	26	2	1	1	0	0	0
HAD	22	3.426	4	2	0.42 (0.21)	0.43 (0.26)	0.12**	22	2	1	1	0	0	0
BRN	22	3.696	4.2	0	0.67 (0.18)	0.54 (0.22)	-0.094*	22	3	2	1.095	1	0.591	0.091
GUR	19	3.185	3.6	1	0.55 (0.24)	0.47 (0.15)	-0.11*	21	3	2	1.208	0	0.867	0.181
JAK	21	3.353	3.8	2	0.59 (0.20)	0.49 (0.15)	-0.158***	22	2	1	1	0	0.591	0
GOU	25	4.29	5.1	4	0.64 (0.22)	0.60 (0.19)	-0.027	25	2	1	1	0	0	0
YUS	26	4.031	4.7	2	0.59 (0.21)	0.57 (0.16)	-0.014	26	6	4	2.397	2	3.253	0.606
MDG	22	3.934	4.4	2	0.61 (0.21)	0.6 (0.15)	0.05	24	2	1	1	0	0	0
Mean	22.1	3.71	4.22	1.85	0.61 (0.22)	0.56 (0.17)	-0.06	23.31		1.39	**1.13**	0.23	0.41	0.07

Number of samples per location (N), allelic richness (A_R_), mean number of alleles per locus per population (NA), observed heterozygosity (H_O_), expected heterozygosity (H_E_), number of private alleles (A_PRIV_), Fixation index (F_IS_), number of alleles at chloroplast SSR loci (N_acpSSR_), number of haplotypes detected in each population (N_b_), effective number of haplotypes (N_e_), number of private haplotypes (Prv), haplotypic richness (Hrs), genetic diversity, (D_V_)

^1^ * p<0.05, ** p<0.01, *** p<0.001.

^**+**^Mean average across all loci with standard deviation.

Genetic diversity parameters and locus level estimates for cpSSR loci are summarised in Tables 2 and S4 respectively. Both cpSSR loci were polymorphic, exhibiting three (ccmp5) and five (ccmp10) alleles per locus among the 303 samples. The combination of alleles resulted in 6 unique haplotypes. Haplotypes H4, H5 and H2 were most frequent occuring in 119, 85 and 79 individuals respectively, whereas H6 was found 14 times and haplotypes H1 and H3 only once. Three haplotypes (H1, H3 and H6) were present in only one population each. Populations YUS harboured two private haplotypes while BRN harboured one private haplotype (Tables 2 and S3). Between one and four haplotypes were found per population, resulting in haplotype richness (H_R_) between 0 and 3.25 (Table 2).

### Population differentiation

**Fig 2:**
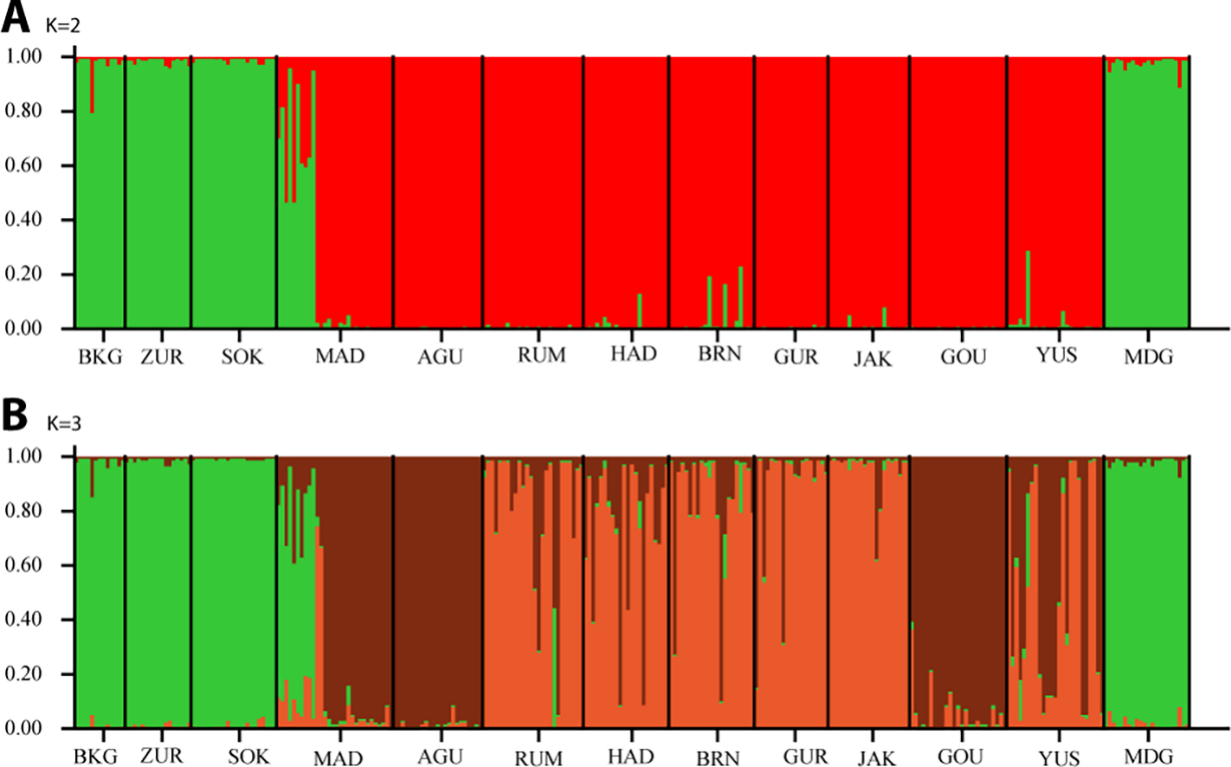
Bar plots of proportional group membership for the 287 individuals genotyped at 10 nSSR loci. **(**A). K = 2 and (B). K = 3. Vertical bars represent samples. Lines separate populations with colours representing the proportion of ancestry derived from each group. Cluster 1 is shown in green, cluster 2 in brown and cluster 3 in orange.

Bayesian analysis of population structure using nSSR suggested the presence of two genetically distinct clusters based on the Evanno method [48], with individuals showing only little mixed ancestry (Fig 2A, S1 Fig). However, K = 3 (Fig 2B) cannot be neglected as it had higher mean likelihood L(K) and still a large value of ΔK. Samples from four geographical populations (BKG, ZUR, SOK and MDG) were affiliated to cluster 1 (green, cluster membership coefficient > 96%). This cluster comprised three populations from the western-most and one from the eastern-most part of the study area. Samples from nine geographical populations made up the red cluster at K = 2 (membership coefficient between 65.4 and 98%), which was again separated at K = 3 into two: cluster 2 (MAD, AGU, GOU–brown) and cluster 3 (RUM, HAD, BRN, GUR, JAK–orange). Population YUS was highly mixed and shared ancestry between clusters 2 and 3 (49% vs 47%). This separation reflected a latitudinal pattern with the northern-most populations making up cluster 2 leaving six populations in the central part of the study area as cluster 3. Population YUS showed mixed ancestry with 49% and 47% affiliation to clusters 2 and 3, respectively (S2 Table).

**Fig 3:**
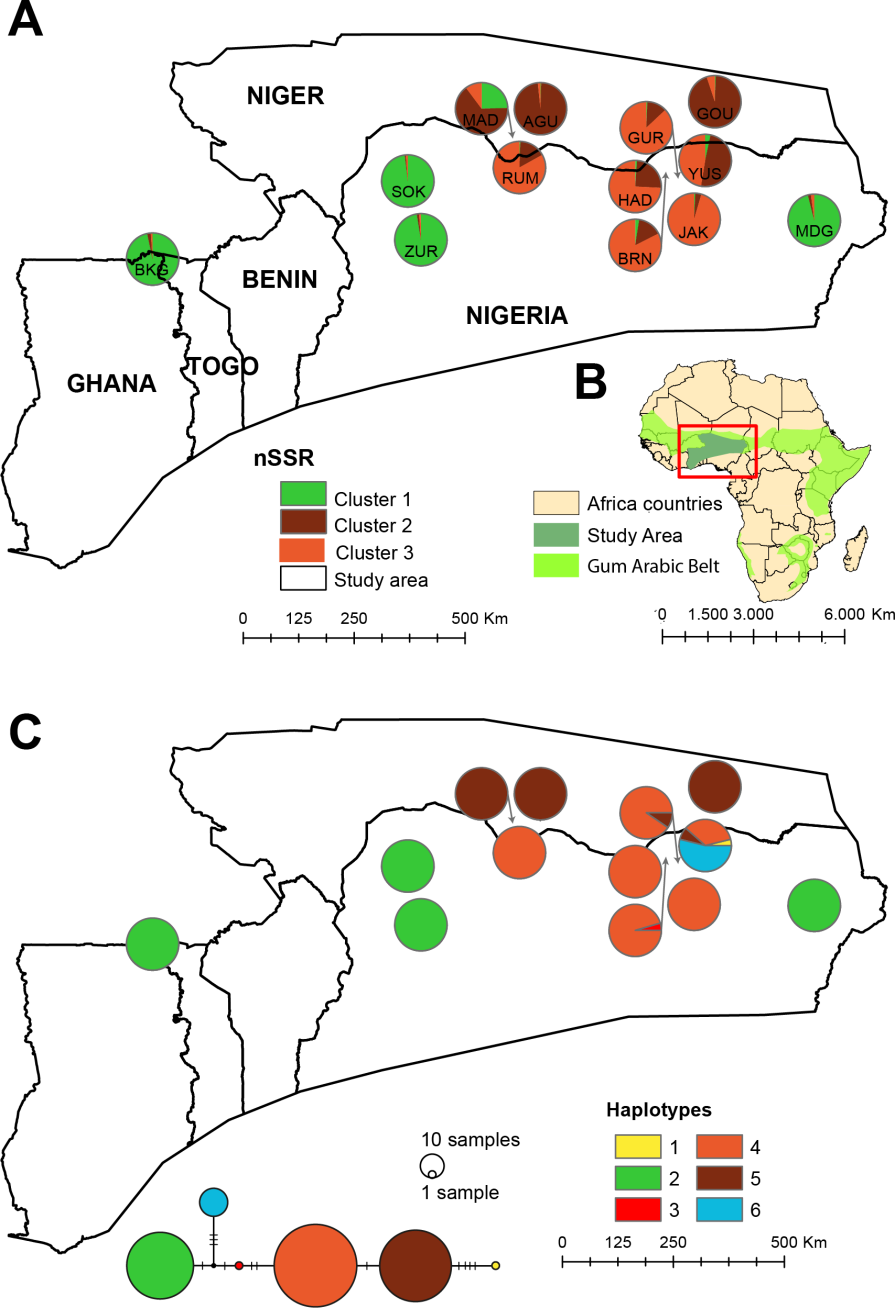
Distribution range, sampling sites and genetic structure for *Senegalia senegal* populations analysed in the present study. (A) nuclear (B) study area and (C) chloroplast genomes. Each population is represented by a pie chart showing proportional membership of clusters or share of haplotypes. Haplotype network was generated by TCS in PopArt with circle sizes proportional to the relative frequency of a particular haplotype.

With respect to cpSSR, 12 out of the 13 populations were either fixed to a single cpDNA haplotype or had a highly dominant haplotype (Fig 3, S3 Table). The population structure at cpSSR loci closely resembled the one at nSSR. While H2 was present in all four populations making up cluster 1, H5 was dominant in populations of cluster 2 and H4 in populations that corresponded to cluster 3. Similar to the nSSR data set, population YUS was mixed of two haplotypes (H4, H6).

With the nuclear data set, non-hierarchical AMOVA revealed 13% variation among and 87% of genetic variation residing within populations (F_ST_ = 0.14; P ≤ 0.001). A hierarchical AMOVA revealed that 18% of the variation resided among clusters at K = 2, while 5% and 77% resided among and within populations (F_ST_ = 0.23; P ≤ 0.001). Considering three clusters, differences among the clusters accounted for 13.5% while variation among and within populations accounted for 4% and 82.5% (F_ST_ = 0.18; P ≤ 0.001) respectively. By contrast, cpSSR non-hierarchical data showed 85% variation among and 15% of genetic variation residing within populations (PhiPT = 0.86; P ≤ 0.001). A hierarchical AMOVA (K = 2) found 51% of the total genetic variance between populations from different clusters (PhiPT = 0.86). Among populations within clusters contributed 39% of the total genetic variance (PhiPR), and 10% of the genetic variation was obtained from within individual populations (PhiPT = 0.89). All three levels contributed significantly to the overall genetic variation as determined through the permutation analyses (Table 3).

**Table 3:** Analysis of molecular variance (AMOVAs) for nSSR and cpDNA in *Senegalia Senegal*.

	nSSR			cpSSR		
Source of variation	DF	% Mol. var.	F-statistics	DF	% Mol. var	Phi-statistics
**Non-hierarchical**						
Among populations	12	13%	F_ST_ = 0.143*	12	85%	
Among individuals within populations	274	0%	F_IS_ = -0.023	290	15%	PhiPT = 0.855*
Within individuals	287	87%	F_IT_ = 0.123*			
**Hierarchical analysis**						
Among clusters (K = 2)	1	18%		1	51%	PhiRT = 0.504*
Among populations within clusters	11	5%	F_ST_ = 0.232*	11	39%	PhiPR = 0.793*
Among individuals within populations	274	0	F_IS_ = -0.023	290	10%	PhiPT = 0.897*
Within individuals	287	77%	F_IT_ = 0.215*			
Among clusters (K = 3)	2	13.50%		2	78%	PhiRT = 0.778*
Among populations within clusters	10	4%	F_ST_ = 0.179*	10	11%	PhiPR = 0.498*
Among individuals within populations	274	0	F_IS_ = -0.023	290	11%	PhiPT = 0.889*
Within individuals	287	82.50%	F_IT_ = 0.160*			

Percentage molecular variance (% Mol. var.), differentiation among individuals (F_ST_), differentiation among individuals within populations (F_IS_), differentiation among populations (F_IT_) is given.

Support is illustrated by a star (*) if p ≤ 0.001.

PhiRT, proportion of the total genetic variance that is due to the variance between clusters; PhiPR, proportion of the total genetic variance that is due to the variance among populations within a cluster; PhiPT, proportion of the total genetic variance that is due to the variance among individuals within a variant.

**Fig 4:**
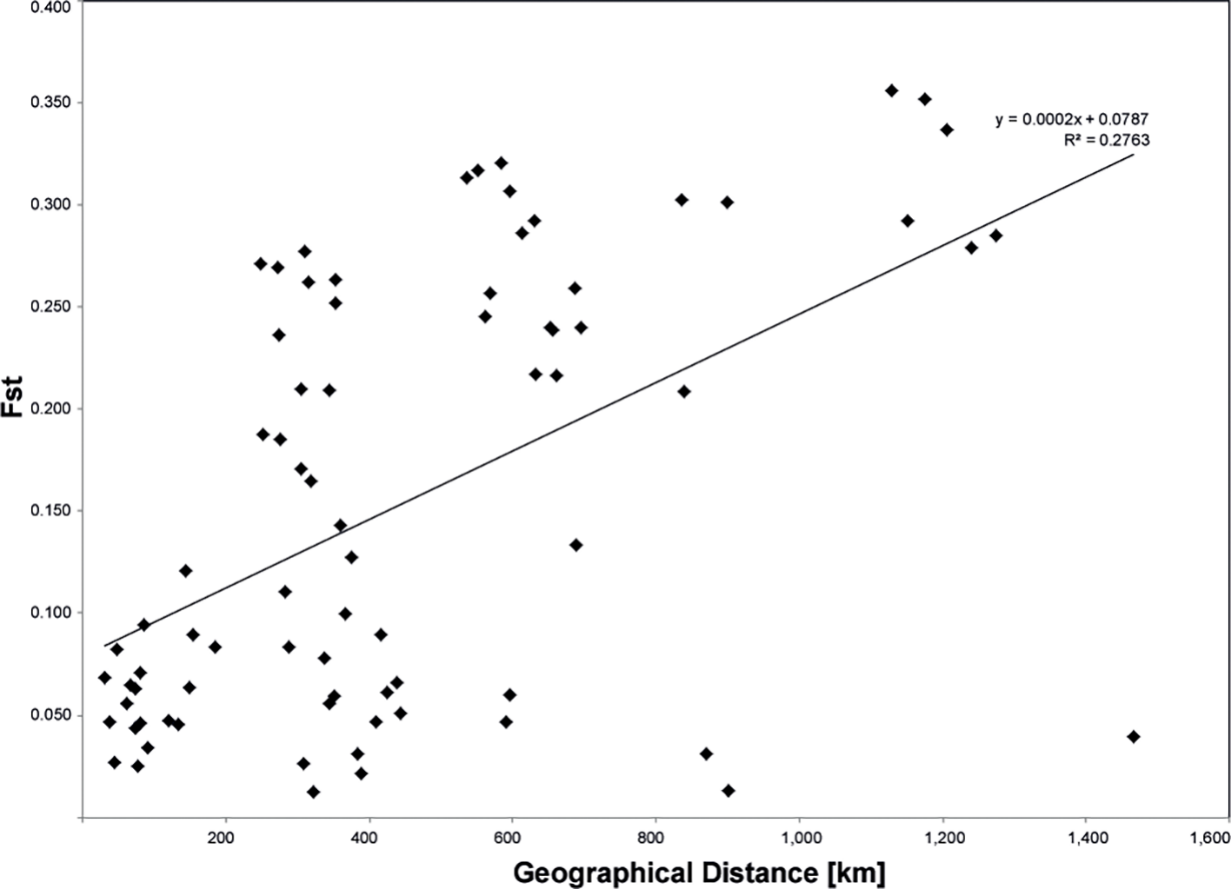
Relationship between genetic and geographic distances (isolation by distance) of *Senegalia senegal* in West Africa.

Pairwise F_ST_ values between populations from different clusters (as suggested by STRUCTURE) were generally higher than those between populations from the same cluster (S6 Table). The Mantel test with 999 permutations revealed that genetic divergence of populations was correlated with geographic distance (r = 0.53; P = 0.001) indicating that the isolation by distance model cannot be rejected (Fig 4). Despite this significant IBD pattern, the geographically very distant but genetically close population MDG within the green nSSR cluster indicates the presence of a larger-scale phylogeographical structure that is not consistent with IBD.

### Distribution modelling

**Fig 5:**
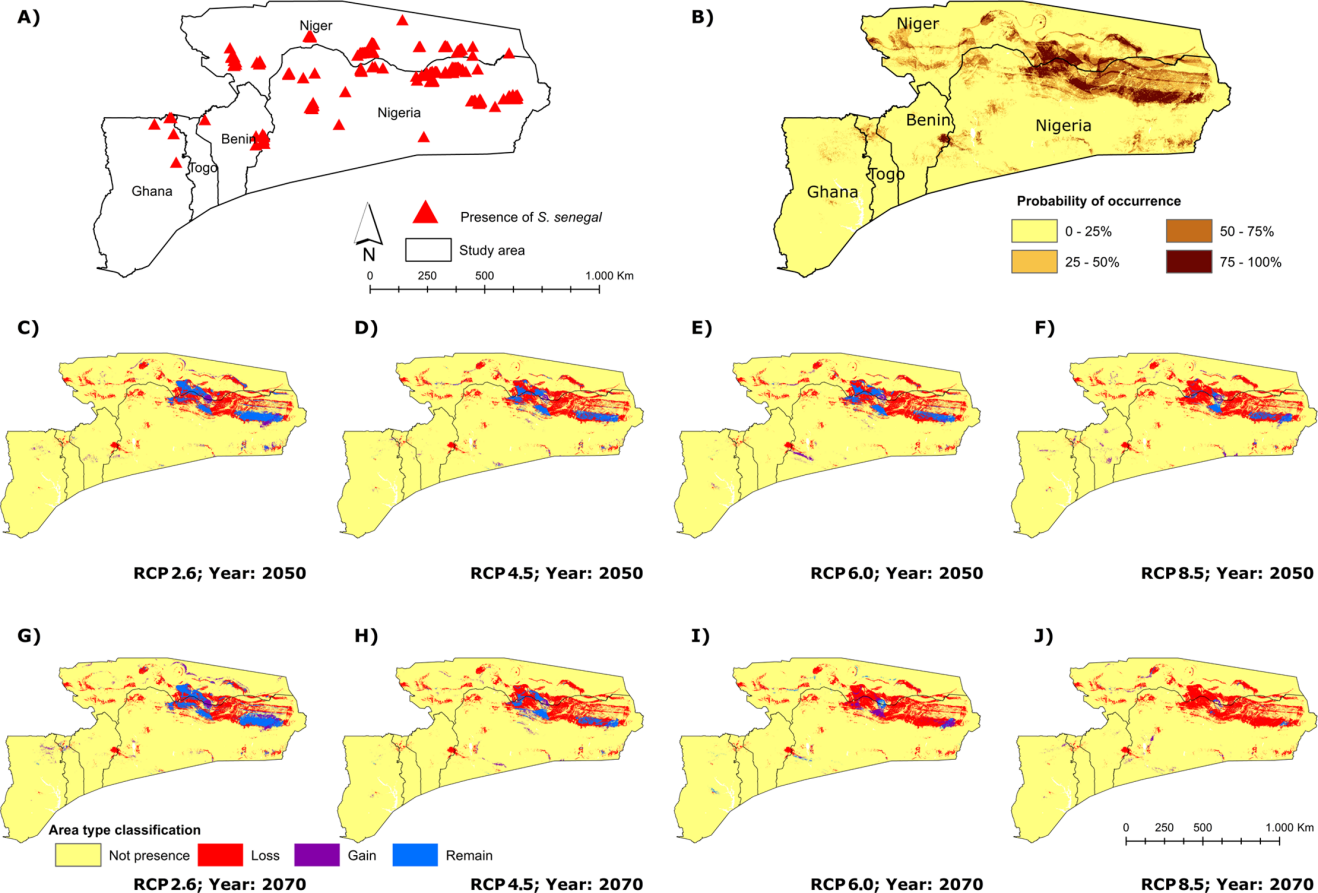
Potential current and future distribution maps for *Senegalia senegal* across the study area. Location of sample points (A, red triangles) and habitat suitability map for *Senegalia senegal* based on present-day climatic conditions (B, brown shaded areas). Predicted potential distribution maps under future conditions: 2050 (C–F) and 2070 (G–J) is given according to the representative concentration pathway climate scenarios. Yellow areas indicate unsuitable conditions for *S*. *senegal*. The numbers identifying each of the RCPs (C–J) refer to the magnitude of the energy imbalance measured in watts per square meters in the scenario in the year under consideration [16].

The final response variable data set to model the current distribution of *Senegalia senegal* included four climatic and three soil variables (Table 4; S5 Table). The MaxEnt model predicting the current distribution (Fig 5A and 5B) was statistically supported with an AUC value of 0.958 (S2 Fig). The distribution of *S*. *senegal* in West Africa is most strongly correlated with extractable nitrogen, coarse fragments, soil organic carbon stock, precipitation of warmest and coldest quarter, precipitation of wettest month and mean temperature of driest quarter (Table 4). Trends in future distribution were similarly forecasted by both GCM but only the resulting maps from GISSER2 are displayed. Our dataset shows that 35.34% to 11.39% of the current potential range of the species will favourably support the species’ ecological requirements across all the scenarios by the year 2050 (Fig 5C–5F). Consequently, by the year 2070, projections show that only 40.96% and 6.34% of the species’ current potential distribution might persist favourably under low emission (RCP 2.6) and high emission (RCP 8.5) scenarios, respectively (Fig 5G–5J). In addition, both GCMs predict a stabilization of habitat loss between 2050 and 2070 under RCP 2.6, while all other scenarios predict additional habitat loss from 2050 to 2070 (S3 Fig).

**Table 4:** Contribution of the seven most important variables to the model.

Ranking	Variable^1^	Importance	Probability of selection
1	Extractable N for 0–30 cm depth	19.19	1.00
2	Coarse fragments at depth 2.0 m	17.93	0.98
3	Soil organic carbon stock at depth 2.0 m	16.24	0.99
4	Precipitation of Warmest Quarter	14.99	1.00
5	Precipitation of Coldest Quarter	14.00	0.95
6	Precipitation of Wettest Month	13.57	0.97
7	Mean Temperature of Driest Quarter	13.14	0.99

## Discussion

### Within-Population genetic diversity

Genetic diversity is a critical measure in population genetics because it provides insights into the current and likely future health of a population [70]. Heterozygosity, described as the average portion of loci with two varying alleles at a single locus within an individual, is a fundamental measure of genetic diversity [71]. Our analyses revealed high levels of genetic diversity within populations of *Senegalia senegal*. Mean expected heterozygosity (H_E_ = 0.56; Table 2) found in this study is comparable to values found in other microsatellite studies of *Senegalia* species (*S*. *senegal*: H_E_ = 0.67, [7]; H_E_ = 0.53, [72]; *S*. *dudgeoni*, H_E_ = 0.6 [72]; *S*. *tortilis*, H_E_ = 0.7, [73]) and other tropical tree species, including *Milicia excelsa* (H_E_ = 0.81, [74]), *Koompassia malaccensis* (H_E_ = 0.85, [75]), and *Vitellaria paradoxa* (H_E_ = 0.73, [76]). Conversely, low levels of genetic diversity can lead to inbreeding depression in the short-term, and to reduced evolutionary potential in the longer term [70, 77].

Half of the investigated sites significantly deviated from HWE. Moderate F_IS_ values were found in one population, indicating a heterozygote deficit. Such a reduction can either be explained by the presence of null alleles [31], the Wahlund effect, or a non-panmictic mating system [72]. As null alleles or Wahlund effects are not evident in our findings, we attribute the deviation from HWE at location HAD (F_IS_ = 0.12) to inbreeding and limited gene-flow consistent with isolation and small population size. On the other hand, heterozygote excess, which was found in six sites, could potentially be due to selection for heterozygosity (i.e. heterozygote advantage), or self-incompatibility and obligate outcrossing [78]. This finding coupled with F_IS_ estimates (mean = 0.06) obtained from our study is in agreement with earlier reports [7, 32] suggesting that *S*. *senegal* is almost exclusively outcrossed and self-incompatible. Studies on morphology and genetic variation of some tropical species showed that outcrossed genotypes also grew faster and had lower mortality than progeny from selfing, resulting in a greater heterozygosity in the populations due to the selective loss of homozygous individuals [78, 79].

Furthermore, Wang et al., [80] reported that a species’ geographic range and its ecological attributes influenced genetic diversity; and that high heterozygosity favoured long-lived plants especially in the arid zones. Our study is congruent with this assumption and is of particular importance when the historical range shifts *Senegalia senegal* had experienced [23], the complex landscapes and fragile ecosystems it occupies [81], and its ecological characteristics, are taken into consideration [82]. Asynchrony in flowering season has been observed for nearby *S*. *senegal* populations (personal observation), which limits inter-population gene flow [83]. Perennial insurgence of wild fires, insect pest invasion, soil erosion, overexploitation and grazing are common ecological challenges throughout these areas [81, 84, 85]. These factors potentially influence or impact gene flow of *S*. *senegal*, as shown for several tropical species [83, 86] and was further supported by Robledo-Arnuncio *et al*. [87]. The impact of these factors on gene flow could have slightly affected the heterozygosity and allele estimates detected in our study, although *S*. *senegal* has been shown to be an outcrossing species [32].

### Population genetic structure and differentiation

There was a strong difference in the extent of genetic structuring revealed by the two types of markers (F_ST_ = 0.14 for nSSRs and F_ST_ = 0.86 for cpSSRs), indicating a much stronger differentiation of the chloroplast genome compared to the nuclear genome. Such differences are commonly observed and are to be expected due to the different types of inheritance and mode of dispersal [88]. Low to moderate values of nuclear population differentiation have previously been documented in *Senegalia senegalia* and were attributed to likely intense gene flow [7, 31, 72]. However, as F_ST_ values only mirror the effects of past events, weak genetic differentiation among populations could also be observed if populations were young, shared a common ancestry and were only weakly differentiated despite lack of current gene flow. The high F_ST_ values in the chloroplast data set suggest that gene flow via seeds is negligible, thus highlighting the importance of pollen vs. seed dispersal for the connectivity of population cohesion in the species [89].

In the analysis of STRUCTURE, two clusters (K = 2), representing a southern and a northern group, may be the most parsimonious explanation for the structure present in the data, yet we consider it not to offer the most biologically meaningful explanation. This is because three clusters (K = 3) better reflect the structure in the data, showing groups of populations that are geographically coherent. Moreover, the three genetic clusters, as revealed by nSSRs were further fully supported by the analysis of cpDNA (Fig 3). Here, the green cluster matched haplotype H2, and the brown and orange clusters matched the closely related haplotypes H5 and H4. Although dispersal via pollen in *Senegalia senegal* might be effective within its natural landscapes coupled with the fact that open low density forest facilitates longer pollen dispersal distances [7], human-mediated dispersal through decades of economic usage might have also influenced the disjunct pattern of populations of cluster 1 observed in the dataset. Because of its agroforestry importance and high quality gum production, *Senegalia* seeds have been traded among nomads, pastorialists and traders of various agricultural products across the region for centuries [25]. Various animals such as cattle, sheep, goat, donkey and camels that graze on the fields for pasture across the entire region play a role in the dispersal of seeds among population. These human-mediated seed transfer is usually along specific routes of similar arid environmental condition, such as MDG, ZUR, and BKG. Frequent gene flow is propagated among those populations without severe interference with populations aside those routes, such as BRN, JAK, and HAD. In addition, sharp environmental transition due to climate change and land use has been shown to be responsible for the increasing aridity from north to south, hence the progressive expansion of *Senegalia* species from the driest to the wettest zones in the SSZ [28]. Consequently, this gradient rather than geographic distance accounts for gene flow among the populations making up cluster 2 and 3 (K = 3), and thus the divergence of populations.

Overall, there is isolation by distance which is in line with gene-flow drift equilibrium and seed dispersal by animals because with geographical clustering, effects of environment cannot be totally excluded. Therefore, isolation-by-environment [90] may have as well contributed to the seemingly disjunct populations. However, because there is congruence in the result obtained from both nSSR and cpSSR, the whole pattern is very likely to be primarily historic (seed dispersal), rather than adaptive, but this study cannot establish if the disjunct pattern is due to isolation by distance or/and isolation by environment.

Odee *et al*. [23], hypothesized a recent east to west range expansion of African populations of *Senegalia senegal* using sequences of ITS and *psbA*. They illustrated that West African *S*. *senegal* was represented by a single genetic group (nuclear), or two haplotypes (chloroplast), respectively. However, the data set in the study was characterized by a low resolution for intraspecific differentiation and contained a sampling gap in the SSZ of West Africa. Our study unveiled more diversity and population structure in *S*. *senegal* (Table 2, Table 3), thus suggesting a more complex pattern than previously reported for this region.

The detected haplotypes, however, showed relationships amongst them and the populations to which they were affiliated. Of particular importance is H6 which is quite distinct appearing in only one population (YUS), with at least six and nine mutations between the two closest dominant relatives (H2 and H4). Although this study does not account for the origin of haplotypes, a colonization event probably from MDG to YUS, or somewhere farther from the east, cannot be neglected.

### Genetic diversity and distribution under climate change

The MaxEnt model confirms the role of soil and climate properties in shaping the distribution of *Senegalia senegal* in the SSZ, as reported by Traore *et al*. [28]. Given the future climate scenarios (Fig 5C–5J), there will be likely a reduction of suitable habitats and population sizes, and potentially a loss of private alleles and private haplotypes leading to declining genetic diversity. All four future climate scenarios vary in magnitude of energy imbalance and the impact of the variation is reflected in the model forecast, as the extent of reduction in suitable range is largely dependent on the species’ ecological requirements. The observed predicted pattern shows a progressive reduction (RCP2.6 > RCP4.5 > RCP6.0 > RCP8.5) of the current potential distribution of *S*. *senegal* within the study area. This shows that *S*. *senegal* may be under threat due to environmental change in the study area and the species might be facing an increased risk of local extinction. However, considering the high allele diversity found in this study, populations may show a predisposition to maintain genetic diversity which may ultimately be a favorable potential for adaptability and persistence should they disperse to track a new suitable range. Consequently, the ability of *S*. *senegal* to persist in an increasingly marginal range will depend on factors such as ecological fitness, genetic make-up and dispersal ability. However, there is no certainty that the dispersal rates of plant species could keep pace with fast rates of environmental change [91].

## Conclusions

Our study unveiled a high genetic and haplotypic variation in the West African Sudano-Sahelian range of *Senegalia senegal* in contrast to previous report that found this part of the range genetically homogenous. We suggest that the species’ colonization history might have played an important role in the distribution of genetic diversity and structuring of populations in the study area. Distribution modeling indicates that depending on the climate scenario, only 41% (RCP 2.6), 35% (RCP 4.5) or down to only 6.3 (RCP 8.5) of suitable area will be available. Consequently, a large part of the geographically structured genetic variation is threatened. In particular the genotypes distributed to the West of the study area, microsatellite cluster 1 and chloroplast haplotype 2, may face strong declines, as hardly any surviving *S*. *senegal* are predicted in this region. Our findings therefore offer insights into how to manage threats or predict responses to disturbances in relation to environmental changes. Finally, the findings of this study will provide a valuable base to reinforce the information available to conservationists and policy makers.

## Supporting information

S1 Table Genetic characteristics of ten nuclear SSR loci in 13 populations of *Senegalia senegal* (*N* = 297). (DOCX)

S2 Table Proportion of membership of each predefined population in each of the inferred clusters at both K = 2 and K = 3. (DOCX)

S3 Table List of haplotypes detected at two cpSSR loci in 13 populations of *Senegalia senegal*. Population YUS is characterized by 2 two unique haplotypes that are not present in any other population. Private haplotypes are highlighted in grey/bold.
(DOCX)

S4 Table Genetic characteristics of two chloroplast microsatellite markers and result of genotyping in *S*. *senegal* (N = 303). (DOCX)

S5 Table List of variables used for modelling the distribution of *Senegalia senegal* in West Africa. (DOCX)

S6 Table Matrix of pairwise *F*_ST_ values based on allele data at ten nuclear (above diagonal) and two chloroplast SSR (above diagonal) loci among 13 populations of *Senegalia senegal*. Most of the values estimated for both marker types were significant (*P* ≤ 0.001). For abbreviations of populations see Table 1.
(DOCX)

S1 Fig Plots for detecting the number of K groups that best fit the data according to [48]. (A) Delta K and (B) Plot of mean likelihood L (K) and variance per K value from STRUCTURE on a dataset containing 287 individuals genotyped for ten nSSR.
(TIF)

S2 Fig Plot of sensitivity vs. specificity. Area under the curve (AUC) value of 0.958 indicates the accuracy of the model prediction.
(TIF)

S3 Fig Predicted area (%) gain or loss of *Senegalia senegal* relative to the current distribution under four future climate scenarios (RCP’s) as estimated by two models (CCSM4, GISSER2) for 2050 and 2070. (TIF)
