# Supplementary material for: Genetic diversity and distribution of Senegalia senegal (L.) Britton under climate change scenarios in West Africa
Source: PLoS One. 2018 Apr 16;13(4):e0194726. doi: 10.1371/journal.pone.0194726 (PMC5901919; doi:10.1371/journal.pone.0194726)
Supplement: S1 Table — (DOCX) [file pone.0194726.s001.docx]

**STable 1. Genetic characteristics of ten nuclear SSR loci in 13 populations of *Senegalia senegal*** (*N* =297)**.**

| **Locus name**^1^ | **NA** | **H_O_** | **H_E_** | **F_IS_** | **F_ST_** | **F_IT_** | **A_R_** | ***P*-val** |
| --- | --- | --- | --- | --- | --- | --- | --- | --- |
| mAsCIRB09 | 7 | 0.322 | 0.209 | -0.070 | 0.075 | 0.010 | 2.230 | ns |
| mAsCIRB10 | 10 | 0.829 | 0.805 | -0.140 | 0.111 | -0.013 | 6.217 | 0.002* |
| mAsCIRC07 | 12 | 0.592 | 0.751 | 0.046 | 0.175 | 0.214 | 6.421 | ns |
| mAsCIRE06 | 11 | 0.606 | 0.692 | 0.062 | 0.084 | 0.141 | 6.252 | 0.002* |
| mAsCIRE07 | 5 | 0.79 | 0.665 | -0.553 | 0.250 | -0.164 | 3.955 | 0.001* |
| mAsCIRE08 | 9 | 0.456 | 0.628 | 0.117 | 0.188 | 0.283 | 5.526 | ns |
| mAsCIRF02 | 12 | 0.49 | 0.656 | 0.062 | 0.174 | 0.226 | 4.853 | ns |
| mAsCIRF03 | 11 | 0.549 | 0.5 | -0.011 | 0.048 | 0.038 | 3.693 | ns |
| mAsCIRH01 | 10 | 0.744 | 0.794 | 0.028 | 0.076 | 0.102 | 6.95 | ns |
| mAsCIRH09 | 13 | 0.626 | 0.638 | -0.075 | 0.297 | 0.245 | 4.535 | ns |
| Total | 110 |  |  |  |  |  | 50.632 |  |
| Mean | 10 | 0.6 | 0.633 | -0.048 | 0.155 | 0.114 | 5.063 |  |

Number of observed alleles per locus (NA), observed heterozygosity (H_O_), expected heterozygosity (H_E_), estimates of F (F_IT_), Theta (F_ST_), and f (F_IS_ ), allelic richness (A_R_), * = Significant deviation from HWE. Support is indicated as p-values (non-significant if p>0.005). ^1^All loci are from Assoumane et al. (2009).
