# Supplementary material for: Genetic diversity and distribution of Senegalia senegal (L.) Britton under climate change scenarios in West Africa
Source: PLoS One. 2018 Apr 16;13(4):e0194726. doi: 10.1371/journal.pone.0194726 (PMC5901919; doi:10.1371/journal.pone.0194726)
Supplement: S2 Table — (DOCX) [file pone.0194726.s002.docx]

**S2 Table.** Proportion of membership of each predefined population in each of the inferred clusters at both K=2 and K=3.

| Given  Pop | (K=2) | | (K=3) | | | Number of  Individuals |
| --- | --- | --- | --- | --- | --- | --- |
|  | Inferred clusters | | Inferred Clusters | | |  |
|  | **I** | II | 1 | 2 | 3 |  |
| BKG | 0.025 | **0.975** | 0.011 | **0.968** | 0.022 | 13 |
| ZUR | 0.013 | **0.987** | 0.012 | **0.975** | 0.013 | 17 |
| SOK | 0.009 | **0.991** | 0.012 | **0.981** | 0.007 | 22 |
| MAD | **0.754** | 0.246 | 0.102 | 0.246 | **0.652** | 30 |
| AGU | **0.995** | 0.005 | 0.017 | 0.005 | **0.979** | 23 |
| RUM | **0.993** | 0.007 | **0.83** | 0.006 | 0.163 | 26 |
| HAD | **0.985** | 0.015 | **0.742** | 0.012 | 0.246 | 22 |
| BRN | **0.966** | 0.034 | **0.824** | 0.025 | 0.15 | 22 |
| GUR | **0.993** | 0.007 | **0.871** | 0.007 | 0.123 | 19 |
| JAK | **0.988** | 0.012 | **0.955** | 0.008 | 0.037 | 21 |
| GOU | **0.994** | 0.006 | 0.051 | 0.006 | **0.942** | 25 |
| YUS | **0.976** | 0.024 | **0.493** | 0.033 | 0.474 | 25 |
| MDG | 0.021 | **0.979** | 0.021 | **0.961** | 0.018 | 22 |

This table show the proportion of membership ancestry of predefined geographical populations across two (K=2) and three (K=3) genetic groups. These estimates represent the fraction of the population’s membership that has ancestry in the subgroup or cluster. Membership coefficient of individuals in each given population is in bold.
