# Supplementary material for: Genetic diversity and distribution of Senegalia senegal (L.) Britton under climate change scenarios in West Africa
Source: PLoS One. 2018 Apr 16;13(4):e0194726. doi: 10.1371/journal.pone.0194726 (PMC5901919; doi:10.1371/journal.pone.0194726)
Supplement: S3 Table — Population YUS is characterized by 2 two unique haplotypes that are not present in any other population. Private haplotypes are highlighted in grey/bold. (DOCX) [file pone.0194726.s003.docx]

**S3 Table**. List of haplotypes detected at two cpSSR loci in 13 populations of *Senegalia senegal*.

| Counts | Haplotype | Haplotype Code | BKG | ZUR | SOK | MAD | AGU | RUM | HAD | BRN | GUR | JAK | GOU | YUS | MDG | Frequency of private haplotype |
| --- | --- | --- | --- | --- | --- | --- | --- | --- | --- | --- | --- | --- | --- | --- | --- | --- |
| 1 | 152 133 | H1 | 0 | 0 | 0 | 0 | 0 | 0 | 0 | 0 | 0 | 0 | 0 | **1** | 0 | 0.038462 |
| 79 | 156 128 | H2 | 13 | 20 | 22 | 0 | 0 | 0 | 0 | 0 | 0 | 0 | 0 | 0 | 24 |  |
| 1 | 156 130 | H3 | 0 | 0 | 0 | 0 | 0 | 0 | 0 | **1** | 0 | 0 | 0 | 0 | 0 | 0.045455 |
| 119 | 156 132 | H4 | 0 | 0 | 0 | 0 | 0 | 26 | 22 | 21 | 19 | 22 | 0 | 9 | 0 |  |
| 89 | 156 133 | H5 | 0 | 0 | 0 | 32 | 28 | 0 | 0 | 0 | 2 | 0 | 25 | 2 | 0 |  |
| 14 | 159 129 | H6 | 0 | 0 | 0 | 0 | 0 | 0 | 0 | 0 | 0 | 0 | 0 | 14 | 0 | 0.538462 |

Population YUS is characterized by two unique haplotypes that are not present in any other population. Private haplotypes are highlighted in grey/bold
