# Supplementary material for: Genetic diversity and distribution of Senegalia senegal (L.) Britton under climate change scenarios in West Africa
Source: PLoS One. 2018 Apr 16;13(4):e0194726. doi: 10.1371/journal.pone.0194726 (PMC5901919; doi:10.1371/journal.pone.0194726)
Supplement: S4 Table — (DOCX) [file pone.0194726.s004.docx]

| **S4 Table.** Genetic characteristics of two chloroplast microsatellite markers and result of genotyping in *Senegalia senegal* (N = 303) | | | | | |
| --- | --- | --- | --- | --- | --- |
| Locus name | Primer Sequence (5'–3') | Allele size (bp) | Repeat Motif | Flourescence | N_acpSSR_ |
| CCMP5 | F:TGTTCCAATATCTTCTTGTCATTT | 152-159 | (C)7 (T)10 (T)5 C (A)11 | NED | 3 |
|  | R: AGGTTCCATCGGAACAATTAT |  |  |  |  |
| CCMP10 | F: TTTTTTTTTAGTGAACGTGTCA | 128-134 | (T)14 | PET | 5 |
|  | R: TTCGTCDDCGTAGTAAATAG |  |  |  |  |

*Notes*: Allele size = expected length of the PCR product in base pairs; N_acpSSR_ = number of alleles per locus at cpSSR loci; Primers published by Weising and Gardner, (1999).
