# Supplementary material for: Genetic diversity and distribution of Senegalia senegal (L.) Britton under climate change scenarios in West Africa
Source: PLoS One. 2018 Apr 16;13(4):e0194726. doi: 10.1371/journal.pone.0194726 (PMC5901919; doi:10.1371/journal.pone.0194726)
Supplement: S5 Table — (DOCX) [file pone.0194726.s005.docx]

**S5 Table**. List of variables used for modelling the distribution of *Senegalia senegal* in West Africa.

| **Code** | **Description** | **Unit** | **Source** |
| --- | --- | --- | --- |
| af_n | Extractable N for 0--30 cm depth | ppm | Hengl et al. 2017 |
| alt | Elevation | m | Hijmans et al. 2005 |
| aspect | Slope aspect | degree | Hijmans et al. 2005 |
| awch3_sl6 | Available soil water capacity for depth 200 cm | % | Hengl et al. 2017 |
| bdticm | Absolute depth to bedrock | cm | Hijmans et al. 2005 |
| bio_1 | Annual Mean Temperature | ºC | Hijmans et al. 2005 |
| bio_2 | Mean Diurnal Range (Mean of monthly (max temp - min temp)) | ºC | Hijmans et al. 2005 |
| bio_3 | Isothermality (BIO2/BIO7) (* 100) |  | Hijmans et al. 2005 |
| bio_4 | Temperature Seasonality (standard deviation *100) | ºC | Hijmans et al. 2005 |
| bio_5 | Max Temperature of Warmest Month | ºC | Hijmans et al. 2005 |
| bio_6 | Min Temperature of Coldest Month | ºC | Hijmans et al. 2005 |
| bio_7 | Temperature Annual Range (BIO5-BIO6) | ºC | Hijmans et al. 2005 |
| bio_8 | Mean Temperature of Wettest Quarter | ºC | Hijmans et al. 2005 |
| bio_9 | Mean Temperature of Driest Quarter | ºC | Hijmans et al. 2005 |
| bio_10 | Mean Temperature of Warmest Quarter | ºC | Hijmans et al. 2005 |
| bio_11 | Mean Temperature of Coldest Quarter | ºC | Hijmans et al. 2005 |
| bio_12 | Annual Precipitation | mm | Hijmans et al. 2005 |
| bio_13 | Precipitation of Wettest Month | mm | Hijmans et al. 2005 |
| bio_14 | Precipitation of Driest Month | mm | Hijmans et al. 2005 |
| bio_15 | Precipitation Seasonality (Coefficient of Variation) | mm | Hijmans et al. 2005 |
| bio_16 | Precipitation of Wettest Quarter | mm | Hijmans et al. 2005 |
| bio_17 | Precipitation of Driest Quarter | mm | Hijmans et al. 2005 |
| bio_18 | Precipitation of Warmest Quarter | mm | Hijmans et al. 2005 |
| bio_19 | Precipitation of Coldest Quarter | mm | Hijmans et al. 2005 |
| bldfie_sl1 | Bulk density (fine earth) at depth 0.00 m | Kg /m3 | Hengl et al. 2017 |
| bldfie_sl2 | Bulk density (fine earth) at depth 0.05 m | Kg /m3 | Hengl et al. 2017 |
| bldfie_sl3 | Bulk density (fine earth) at depth 0.15 m | Kg /m3 | Hengl et al. 2017 |
| bldfie_sl4 | Bulk density (fine earth) at depth 0.30 m | Kg /m3 | Hengl et al. 2017 |
| bldfie_sl5 | Bulk density (fine earth) at depth 0.60 m | Kg /m3 | Hengl et al. 2017 |
| bldfie_sl6 | Bulk density (fine earth) at depth 1.00 m | Kg /m3 | Hengl et al. 2017 |
| bldfie_sl7 | Bulk density (fine earth) at depth 2.00 m | Kg /m3 | Hengl et al. 2017 |
| cecsol_sl1 | Cation exchange capacity of soil at depth 0.00 m | cmolc/kg | Hengl et al. 2017 |
| cecsol_sl2 | Cation exchange capacity of soil at depth 0.05 m | cmolc/kg | Hengl et al. 2017 |
| cecsol_sl3 | Cation exchange capacity of soil at depth 0.15 m | cmolc/kg | Hengl et al. 2017 |
| cecsol_sl4 | Cation exchange capacity of soil at depth 0.30 m | cmolc/kg | Hengl et al. 2017 |
| cecsol_sl5 | Cation exchange capacity of soil at depth 0.60 m | cmolc/kg | Hengl et al. 2017 |
| cecsol_sl6 | Cation exchange capacity of soil at depth 1.00 m | cmolc/kg | Hengl et al. 2017 |
| cecsol_sl7 | Cation exchange capacity of soil at depth 2.00 m | cmolc/kg | Hengl et al. 2017 |
| clyppt_sl1 | Clay content (0-2 micro meter) at depth 0.00 m | % | Hengl et al. 2017 |
| clyppt_sl2 | Clay content (0-2 micro meter) at depth 0.05 m | % | Hengl et al. 2017 |
| clyppt_sl3 | Clay content (0-2 micro meter) at depth 0.15 m | % | Hengl et al. 2017 |
| clyppt_sl4 | Clay content (0-2 micro meter) at depth 0.30 m | % | Hengl et al. 2017 |
| clyppt_sl5 | Clay content (0-2 micro meter) at depth 0.60 m | % | Hengl et al. 2017 |
| clyppt_sl6 | Clay content (0-2 micro meter) at depth 1.00 m | % | Hengl et al. 2017 |
| clyppt_sl7 | Clay content (0-2 micro meter) at depth 2.00 m | % | Hengl et al. 2017 |
| crfvol_sl1 | Coarse fragments at depth 0.00 m | % | Hengl et al. 2017 |
| crfvol_sl2 | Coarse fragments at depth 0.05 m | % | Hengl et al. 2017 |
| crfvol_sl3 | Coarse fragments at depth 0.15 m | % | Hengl et al. 2017 |
| crfvol_sl4 | Coarse fragments at depth 0.30 m | % | Hengl et al. 2017 |
| crfvol_sl5 | Coarse fragments at depth 0.60 m | % | Hengl et al. 2017 |
| crfvol_sl6 | Coarse fragments at depth 1.00 m | % | Hengl et al. 2017 |
| crfvol_sl7.1 | Coarse fragments at depth 2.00 m | % | Hengl et al. 2017 |
| ocstha_sl1 | Soil organic carbon stock at depth 0.00 m | tn/ha | Hengl et al. 2017 |
| ocstha_sl2 | Soil organic carbon stock at depth 0.05 m | tn/ha | Hengl et al. 2017 |
| ocstha_sl3 | Soil organic carbon stock at depth 0.15 m | tn/ha | Hengl et al. 2017 |
| ocstha_sl4 | Soil organic carbon stock at depth 0.30 m | tn/ha | Hengl et al. 2017 |
| ocstha_sl5 | Soil organic carbon stock at depth 0.60 m | tn/ha | Hengl et al. 2017 |
| ocstha_sl6 | Soil organic carbon stock at depth 1.00 m | tn/ha | Hengl et al. 2017 |
| ocstha_sl7.1 | Soil organic carbon stock at depth 2.00 m | tn/ha | Hengl et al. 2017 |
| phhihox_sl1 | Soil pH in H2O at depth 0.00 m | -- | Hengl et al. 2017 |
| phhihox_sl2 | Soil pH in H2O at depth 0.05 m | -- | Hengl et al. 2017 |
| phhihox_sl3 | Soil pH in H2O at depth 0.15 m | -- | Hengl et al. 2017 |
| phhihox_sl4 | Soil pH in H2O at depth 0.30 m | -- | Hengl et al. 2017 |
| phhihox_sl5 | Soil pH in H2O at depth 0.60 m | -- | Hengl et al. 2017 |
| phhihox_sl6 | Soil pH in H2O at depth 1.00 m | -- | Hengl et al. 2017 |
| phhihox_sl7 | Soil pH in H2O at depth 2.00 m | -- | Hengl et al. 2017 |
| phhikcl_sl1 | Soil pH in KCl at depth 0.00 m | -- | Hengl et al. 2017 |
| phhikcl_sl2 | Soil pH in KCl at depth 0.05 m | -- | Hengl et al. 2017 |
| phhikcl_sl3 | Soil pH in KCl at depth 0.15 m | -- | Hengl et al. 2017 |
| phhikcl_sl4 | Soil pH in KCl at depth 0.30 m | -- | Hengl et al. 2017 |
| phhikcl_sl5 | Soil pH in KCl at depth 0.60 m | -- | Hengl et al. 2017 |
| phhikcl_sl6 | Soil pH in KCl at depth 1.00 m | -- | Hengl et al. 2017 |
| phhikcl_sl7 | Soil pH in KCl at depth 2.00 m | -- | Hengl et al. 2017 |
| slope | Slope Steepness | % | Hengl et al. 2017 |
| sltppt_sl1 | Silt content (2-50 micro meter) at depth 0.00 m | % | Hengl et al. 2017 |
| sltppt_sl2 | Silt content (2-50 micro meter) at depth 0.05 m | % | Hengl et al. 2017 |
| sltppt_sl3 | Silt content (2-50 micro meter) at depth 0.15 m | % | Hengl et al. 2017 |
| sltppt_sl4 | Silt content (2-50 micro meter) at depth 0.30 m | % | Hengl et al. 2017 |
| sltppt_sl5 | Silt content (2-50 micro meter) at depth 0.60 m | % | Hengl et al. 2017 |
| sltppt_sl6 | Silt content (2-50 micro meter) at depth 1.00 m | % | Hengl et al. 2017 |
| sltppt_sl7 | Silt content (2-50 micro meter) at depth 2.00 m | % | Hengl et al. 2017 |
| sndppt_sl1 | Sand content (50-2000 micro meter) at depth 0.00 m | % | Hengl et al. 2017 |
| sndppt_sl2 | Sand content (50-2000 micro meter) at depth 0.05 m | % | Hengl et al. 2017 |
| sndppt_sl3 | Sand content (50-2000 micro meter) at depth 0.15 m | % | Hengl et al. 2017 |
| sndppt_sl4 | Sand content (50-2000 micro meter) at depth 0.30 m | % | Hengl et al. 2017 |
| sndppt_sl5 | Sand content (50-2000 micro meter) at depth 0.60 m | % | Hengl et al. 2017 |
| sndppt_sl6 | Sand content (50-2000 micro meter) at depth 1.00 m | % | Hengl et al. 2017 |
| sndppt_sl7 | Sand content (50-2000 micro meter) at depth 2.00 m | % | Hengl et al. 2017 |
