# Supplementary material for: Genetic diversity and distribution of Senegalia senegal (L.) Britton under climate change scenarios in West Africa
Source: PLoS One. 2018 Apr 16;13(4):e0194726. doi: 10.1371/journal.pone.0194726 (PMC5901919; doi:10.1371/journal.pone.0194726)
Supplement: S6 Table — Most of the values estimated for both marker types were significant (P ≤ 0.001). For abbreviations of populations see Table 1. (DOCX) [file pone.0194726.s006.docx]

**S6 Table.** Matrix of pairwise *F*_ST_ values based on allele data at ten nuclear (above diagonal) and two chloroplast SSR (above diagonal) loci among 13 populations of *Senegalia senegal*. Most of the values estimated for both marker types were significant (*P* ≤ 0.05; *P* ≤ 0.001). For abbreviations of populations see Table 1.

|  | BKG | ZUR | SOK | MAD | AGU | RUM | HAD | BRN | GUR | JAK | GOU | YUS | MDG |
| --- | --- | --- | --- | --- | --- | --- | --- | --- | --- | --- | --- | --- | --- |
| BKG | - | 0.0599 | 0.0467 | 0.2084 | 0.3012 | 0.3024 | 0.3557 | 0.2925 | 0.3517 | 0.3369 | 0.2851 | 0.2792 | 0.0397 |
| ZUR | 0 | - | 0.0453 | 0.1706 | 0.2636 | 0.2695 | 0.313 | 0.2454 | 0.3204 | 0.286 | 0.2398 | 0.2398 | 0.0312 |
| SOK | 0 | 0 | - | 0.1878 | 0.2772 | 0.2713 | 0.3169 | 0.257 | 0.3065 | 0.2921 | 0.2593 | 0.2389 | 0.0128 |
| MAD | 0.061417 | 0.061417 | 0.061417 | - | 0.0557 | 0.0648 | 0.0779 | 0.0559 | 0.1273 | 0.0896 | 0.0508 | 0.0611 | 0.1336 |
| AGU | 0.061417 | 0.061417 | 0.061417 | 0 | - | 0.0944 | 0.1103 | 0.0837 | 0.1648 | 0.1432 | 0.0313 | 0.0999 | 0.2169 |
| RUM | 0.069245 | 0.069245 | 0.069245 | 0.065829 | 0.065829 | - | 0.0262 | 0.0126 | 0.0592 | 0.0214 | 0.0662 | 0.0465 | 0.2167 |
| HAD | 0.069245 | 0.069245 | 0.069245 | 0.065829 | 0.065829 | 0 | - | 0.047 | 0.0823 | 0.046 | 0.0832 | 0.0476 | 0.2522 |
| BRN | 0.063083 | 0.063083 | 0.063083 | 0.060112 | 0.060112 | 0.000147 | 0.000147 | - | 0.0685 | 0.0253 | 0.0634 | 0.0341 | 0.2089 |
| GUR | 0.058463 | 0.058463 | 0.058463 | 0.049974 | 0.049974 | 0.000624 | 0.000624 | 0.000446 | - | 0.0266 | 0.1209 | 0.0631 | 0.2623 |
| JAK | 0.069245 | 0.069245 | 0.069245 | 0.065829 | 0.065829 | 0 | 0 | 0.000147 | 0.000624 | - | 0.0897 | 0.0437 | 0.2365 |
| GOU | 0.061417 | 0.061417 | 0.061417 | 0 | 0 | 0.065829 | 0.065829 | 0.060112 | 0.049974 | 0.065829 | - | 0.071 | 0.2095 |
| YUS | 0.03462 | 0.03462 | 0.03462 | 0.029771 | 0.029771 | 0.018705 | 0.018705 | 0.016656 | 0.01468 | 0.018705 | 0.029771 | - | 0.1852 |
| MDG | 0 | 0 | 0 | 0.061417 | 0.061417 | 0.069245 | 0.069245 | 0.063083 | 0.058463 | 0.069245 | 0.061417 | 0.03462 | - |
