# Supplementary figures and images for: Genetic diversity and distribution of Senegalia senegal (L.) Britton under climate change scenarios in West Africa
Source: PLoS One. 2018 Apr 16;13(4):e0194726. doi: 10.1371/journal.pone.0194726 (PMC5901919; doi:10.1371/journal.pone.0194726)

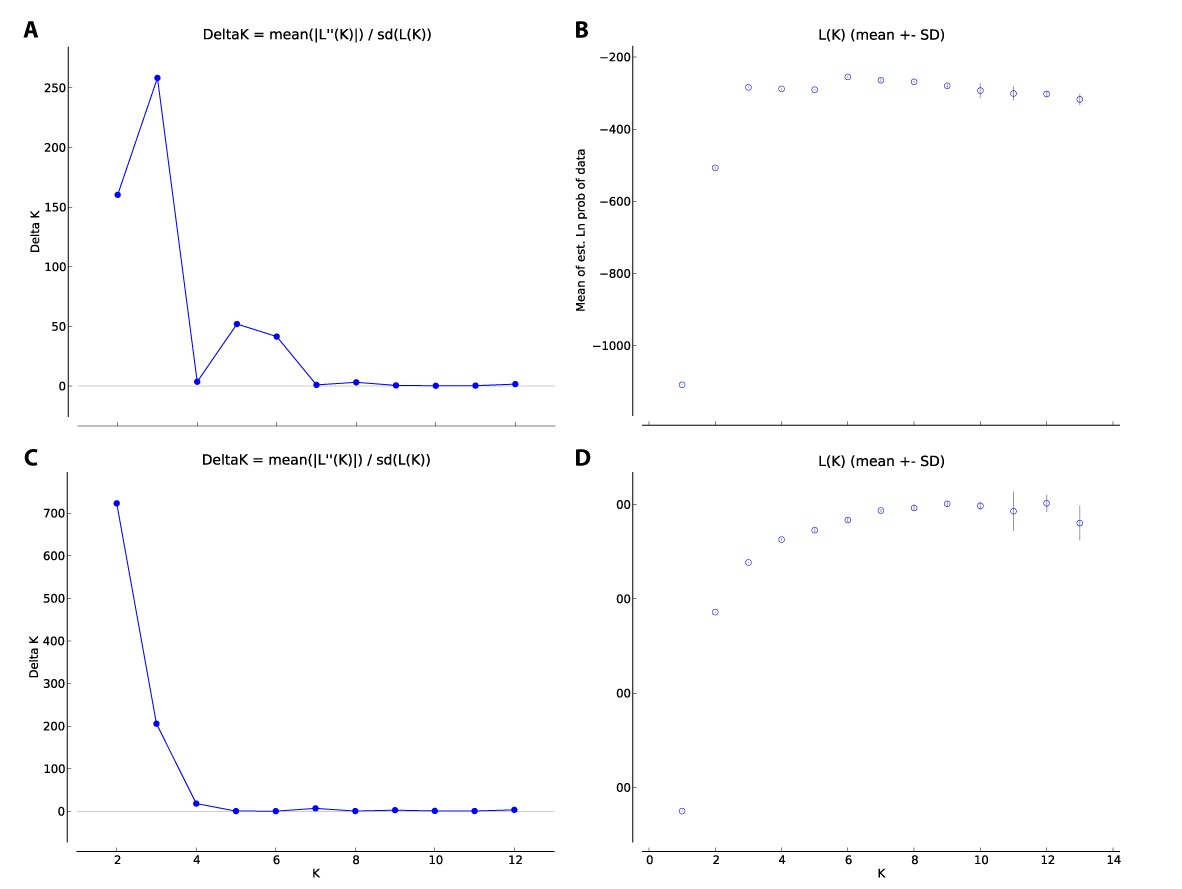

Supplement: S1 Fig — (A) Delta K and (B) Plot of mean likelihood L (K) and variance per K value from STRUCTURE on a dataset containing 287 individuals genotyped for ten nSSR. (TIF) [file pone.0194726.s007.tif]

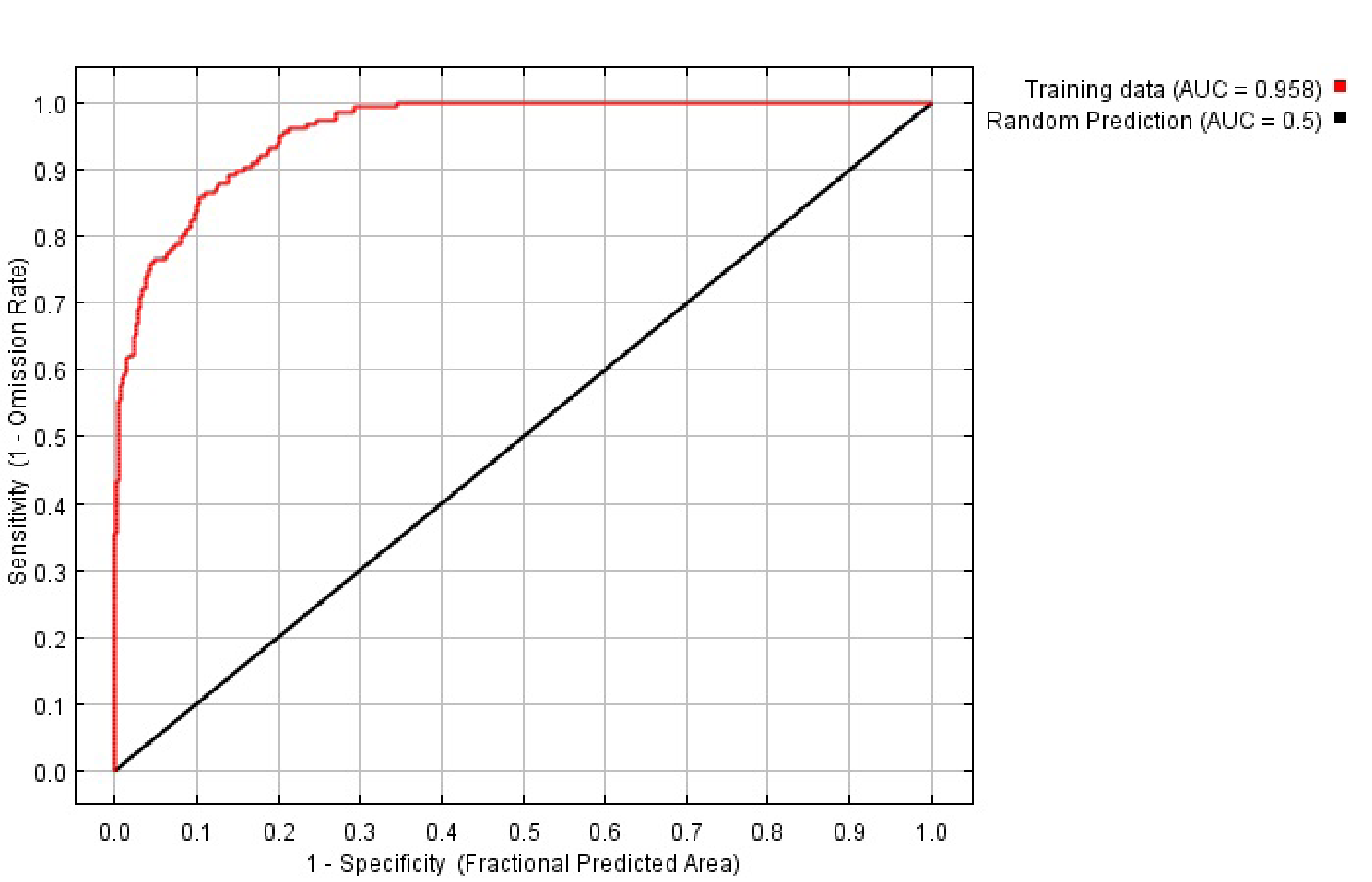

Supplement: S2 Fig — Area under the curve (AUC) value of 0.958 indicates the accuracy of the model prediction. (TIF) [file pone.0194726.s008.tif]

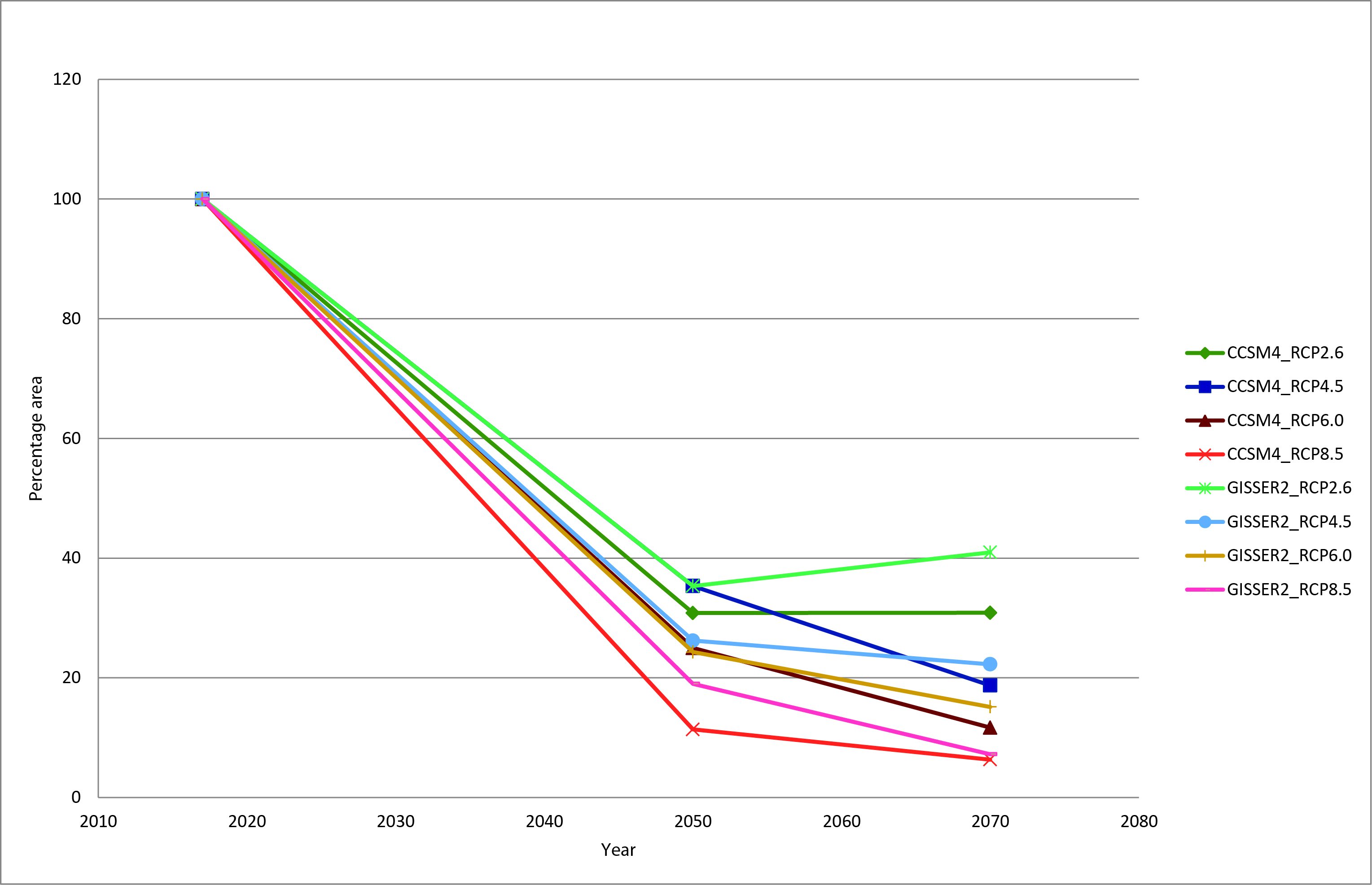

Supplement: S3 Fig — (TIF) [file pone.0194726.s009.tif]
